# Magnitude integration in the Archerfish

**DOI:** 10.1038/s41598-021-94956-1

**Published:** 2021-08-02

**Authors:** Tali Leibovich-Raveh, Ashael Raveh, Dana Vilker, Shai Gabay

**Affiliations:** 1 Department of Mathematics Education, Faculty of Education, University of Haifa, Haifa, Israel; 2 The Institute of Information Processing and Decision Making and the School of Psychological Sciences, University of Haifa, Haifa, Israel; 3 Department of Evolutionary and Environmental Biology, Faculty of Natural Sciences, University of Haifa, Haifa, Israel

**Keywords:** Decision, Perception, Animal behaviour

## Abstract

We make magnitude-related decisions every day, for example, to choose the shortest queue at the grocery store. When making such decisions, which magnitudes do we consider? The dominant theory suggests that our focus is on numerical quantity, i.e., the number of items in a set. This theory leads to quantity-focused research suggesting that discriminating quantities is automatic, innate, and is the basis for mathematical abilities in humans. Another theory suggests, instead, that non-numerical magnitudes, such as the total area of the compared items, are usually what humans rely on, and numerical quantity is used only when required. Since wild animals must make quick magnitude-related decisions to eat, seek shelter, survive, and procreate, studying which magnitudes animals spontaneously use in magnitude-related decisions is a good way to study the relative primacy of numerical quantity versus non-numerical magnitudes. We asked whether, in an animal model, the influence of non-numerical magnitudes on performance in a spontaneous magnitude comparison task is modulated by the number of non-numerical magnitudes that positively correlate with numerical quantity. Our animal model was the Archerfish, a fish that, in the wild, hunts insects by shooting a jet of water at them. These fish were trained to shoot water at artificial targets presented on a computer screen above the water tank. We tested the Archerfish's performance in spontaneous, untrained two-choice magnitude decisions. We found that the fish tended to select the group containing larger non-numerical magnitudes and smaller quantities of dots. The fish selected the group containing more dots mostly when the quantity of the dots was positively correlated with all five different non-numerical magnitudes. The current study adds to the body of studies providing direct evidence that in some cases animals’ magnitude-related decisions are more affected by non-numerical magnitudes than by numerical quantity, putting doubt on the claims that numerical quantity perception is the most basic building block of mathematical abilities.

## Introduction

Across the animal kingdom, humans and other animals must quickly make magnitude-related decisions to survive. For example, for fish, joining the larger school of fish may provide additional protection against predators and hence may be the difference between life and death. How do animals make such decisions? Until recently, the dominant theory suggested that all animals have an innate sense of number^1–3^. Accordingly, in such decisions, the fish in our example will roughly estimate the number of fish in each school when deciding which school to join. This decision will be based solely on numerical quantity. Yet other theories suggest that such a decision may require integrating information available for multiple magnitudes, both numerical and non-numerical, such as the space taken by the fish, their density, etc., to reach a decision^4–6^.

In that context, one of the most significant current discussions is whether, in such magnitude-related decisions as described above, discrete numerical quantity (i.e., the number of items in a set) is extracted from a set of items automatically, intuitively, and early in the processing stage^1–3^, or whether non-numerical magnitudes are extracted early and automatically, while numerical quantity is extracted later in the process, in a more purposeful and effortful way, and only when the task calls for it^4–6^.

Since animals are required to make quick magnitude-related decisions to eat, seek shelter, survive and procreate, studying which magnitudes animals spontaneously use in magnitude-related decisions e.g.,^7–14^, is a good way to study the relative primacy of numerical quantity versus non-numerical magnitudes. Studies that employ this approach share some commonalities. First, they usually use biologically relevant stimuli. For example, schools of fish (to ask which school a fish would prefer to join to survive), pieces of food (to ask which cluster of food the animal would prefer^15^), or stimuli that were previously associated with food^16,17^, etc. Second, these studies usually use a small number of items, ranging from 1 to 20, but mostly up to 8 items per group. Third, and most important, most of these studies focus on whether the animal can discriminate between numerical quantities while attempting to control for the possible influence of non-numerical magnitudes. 'Controlling' non-numerical magnitudes is problematic; it is impossible to have two groups of items that differ only in number, but not in non-numerical magnitudes (for a detailed discussion please see^6^). For example, Stancher et al.^18^ asked whether frogs can spontaneously discriminate between quantities (1–8 larvae), and reported that frogs were able to discriminate between quantities when 1 was compared to small quantities. In contrast, with higher quantities frogs may have used either numerical quantity or non-numerical magnitudes. In the study, movement, volume, total surface, and weight were controlled (e.g., overall total surface similar in both the to-be-compared groups of larvae). Importantly, some of the controls were made when the numerical quantity 1 was compared to 2 or more. Hence, there may be a specific preference to 1 versus ‘many’ or vice versa. Second, it is unclear whether and how other non-numerical magnitudes that are not mentioned in the manuscript were controlled, such as density or convex hull for example. In another example, Yang et al.^8^ asked whether cuttlefish can discriminate between different numerical quantities of prey (shrimps). In one experiment, all the shrimps in the two plates the fish had to choose between, were of the same size. Therefore, the plate with the larger number of shrimps also contained a larger surface area of shrimps. In a follow-up experiment, the authors equated the density of the shrimps on each plate. However, this meant that the larger number of shrimps was spread over a larger area, so in these cases, the convex hull was correlated with the number of shrimps. The authors concluded that cuttlefish could discriminate between numerical quantities, although an alternative explanation that was not ruled out is that the fish used total surface area or convex hull to choose the larger group.

Sometimes, the attempt to reduce the influence of non-numerical magnitudes creates a situation where the variability of the numerical quantity is small (only a small number of quantities are used), while the variability in non-numerical magnitude is much greater. For example, in an attempt to test whether chicks can discriminate 5 versus 10 or 10 versus 20, Rugani et al.^16^ randomized the size of the elements presented to the chicks. While there is not enough information about the condition such randomization of size created, the variability of non-numerical magnitudes was much higher than the variability of numerical quantity, which could make numerical quantity the more salient magnitude in this situation.

Importantly, some studies have demonstrated that animals use non-numerical magnitudes alone, or a combination of numerical and non-numerical magnitudes, in magnitude comparison tasks. Sometimes different studies with the same species reported contradicting findings. For example, in 2011, Gomez-Laplaza and Gerlai^19^ concluded that angelfish can discriminate between different numerical quantities of same-size food items, based on the *number* of food pieces. Recently, however, the same research group^14^ repeated the experiment in three different versions: when only one item of food was presented on each plate, the fish have shown a preference for the larger item. When the number of food items was different but the total surface area was equal, fish preferred the plate where the food items were physically larger. When numerical quantity and size were negatively correlated, namely, the larger the amount, the smaller the food, the fish have shown no preference. Therefore, the authors concluded that non-numerical magnitudes also play an important role when angelfish makes magnitude-related decisions. Similar conclusions as to the importance of non-numerical magnitudes were found in different animals, such as crickets^12^, large carnivores^10^, amphibians^20^, and fish^21^.

To summarize, the picture emerging from previous findings is that many species of animals spontaneously use both numerical quantity and non-numerical magnitudes to make a magnitude-related decision that may greatly impact their survival. These studies usually employ relatively small quantities, and biologically relevant stimuli, and were limited in their ability to account for the influence of non-numerical magnitudes. In addition, the spotlight in most studies was directed towards numerical quantities, and non-numerical magnitudes were more often 'controlled' than studied.

Against this background, we would like to turn the spotlight to non-numerical magnitudes, and systematically study how both numerical quantities and non-numerical magnitudes may influence the decision in a spontaneous magnitude comparison task, when the relationship between numerical quantities and non-numerical magnitudes is manipulated within the same block and the same subjects.

More specifically, we asked whether the influence of non-numerical magnitudes on performance in a spontaneous magnitude comparison task is modulated by the number of non-numerical magnitudes that positively correlate with numerical quantity. A similar question was investigated by Leibovich and Ansari^22^. In this study, human adults were asked to select the group comprised of more dots, while their brains were being scanned in an MRI machine. In addition to the number of dots, five non-numerical magnitudes were recorded: The total surface area of the dots, their average diameter, total circumference, density, and convex hull (the total area of the dots and the space between them). The authors manipulated the number of non-numerical magnitudes that were congruent (i.e., positively correlate) or incongruent (i.e., negatively correlate) with numerical quantity; in congruity level one, only one non-numerical magnitude was congruent with numerical quantity and the remaining four non-numerical magnitudes were incongruent with numerical quantity. In congruity level four, four out of five non-numerical magnitudes were congruent with numerical quantity. The activity of the right inferior frontal gyrus (rIFG) positively correlated with the level of congruity. Accordingly, the authors suggested that the rIFG supports the accumulation of non-numerical magnitudes that positively correlates with numerical quantity in such comparison tasks.

In the current study, we tested the Archerfish's (*Toxotes sp.*) performance in spontaneous, untrained two-choice magnitude decisions, using a similar approach to the one employed in Leibovich and Ansari's study^22^. This allowed us to add to the current body of knowledge in several ways. First, we used stimuli that are not biologically relevant (groups of dots), allowing us more control over the non-numerical properties of our stimuli. In addition, this is as close as we could have got to test untrained magnitude preference in general, and not in a specific biological context such as foraging or shelter seeking, which might be influenced by instinctual behaviors elicited by the biologically relevant context. With that being said, we still use the hunting response of the fish for this task (shooting water at the stimuli), and the fish are being rewarded by food. For this reason, the study is not completely context-free. Second, we used a larger number of items (between 5–35 per group), thereby expanding the discussion to larger quantities. Third, since in congruity levels 2 and 4, different non-numerical magnitudes were manipulated (see Table 1), we were also able to ask whether it is the number of non-numerical magnitudes, or some specific non-numerical magnitudes, that influence performance.

**Table 1:** Different congruity levels.

Congruity level	Convex hull	Density	Total surface	Average diameter	Total circumference
1	IC	IC	IC	IC	**C**
2a	**C**	IC	IC	IC	**C**
2b	IC	**C**	IC	IC	**C**
3	**C**	IC	**C**	IC	**C**
4a	IC	**C**	**C**	**C**	**C**
4b	**C**	**C**	**C**	IC	**C**
5	**C**	**C**	**C**	**C**	**C**

*C* congruent with number, *IC* incongruent with number.

The congruent magnitudes are in bold font.

Ray-finned fish and mammals diverged more than 400 million years ago. Therefore, studying fish can teach us which behaviors already existed early in the evolutionary process and were conserved throughout it, or whether a behavior may have been important enough to emerge independently in different species^23^. Archerfish in the wild hunt by shooting a jet of water at insects, causing them to fall into the water^23^. These fish can be trained to respond to artificial targets presented on a computer monitor in an experimental setting. Accordingly, they can be used as the fish equivalent of a human subject reporting psychophysical decisions by pressing a key^24,25^.

Archerfish do not possess any brain structures homologous to the mammalian frontal or parietal cortex that are known to be involved in numerical cognition. This does not rule out the existence of an analogous structure, though. A recent study by Messina et al.^26^ examined the brain structures involved with size and number perception in Zebrafish. The fish were habituated to either numerical quantity, shape, or size. Following the habituation, the expression of genes in response to a regulatory signal (i.e., immediate early genes) was tested in several brain regions. In some brain regions, i.e., the telencephalon and thalamus, the modulation of the tested immediate early genes was mainly the result of habituation to numerical quantity. In other regions, i.e., the retina and the optic tectum (which are earlier in the visual pathway), the modulation of the tested immediate early genes was mainly the result of habituation to changes in physical size, namely, the total surface area. Although the Zebrafish brain and the Archerfish brain are different, the study of Messina et al. is a great demonstration of magnitude processing in brain regions not homologous to the cortex, and that non-numerical magnitudes are processed by more primary regions than numerical quantity.

Nevertheless, it seems that the hunting method of the Archerfish requires some degree of basic magnitude discrimination; because hunting requires the fish to come up to the surface of the water where they are exposed to predators, and because insects can fly out of reach at any moment, the fish must quickly choose which insects to hunt. In such decisions, it is conceivable that different non-numerical magnitudes, such as the physical size of the insect or the number of insects in the same area, can be among the factors Archerfish take into consideration during hunting.

## Results

**Figure 1:**
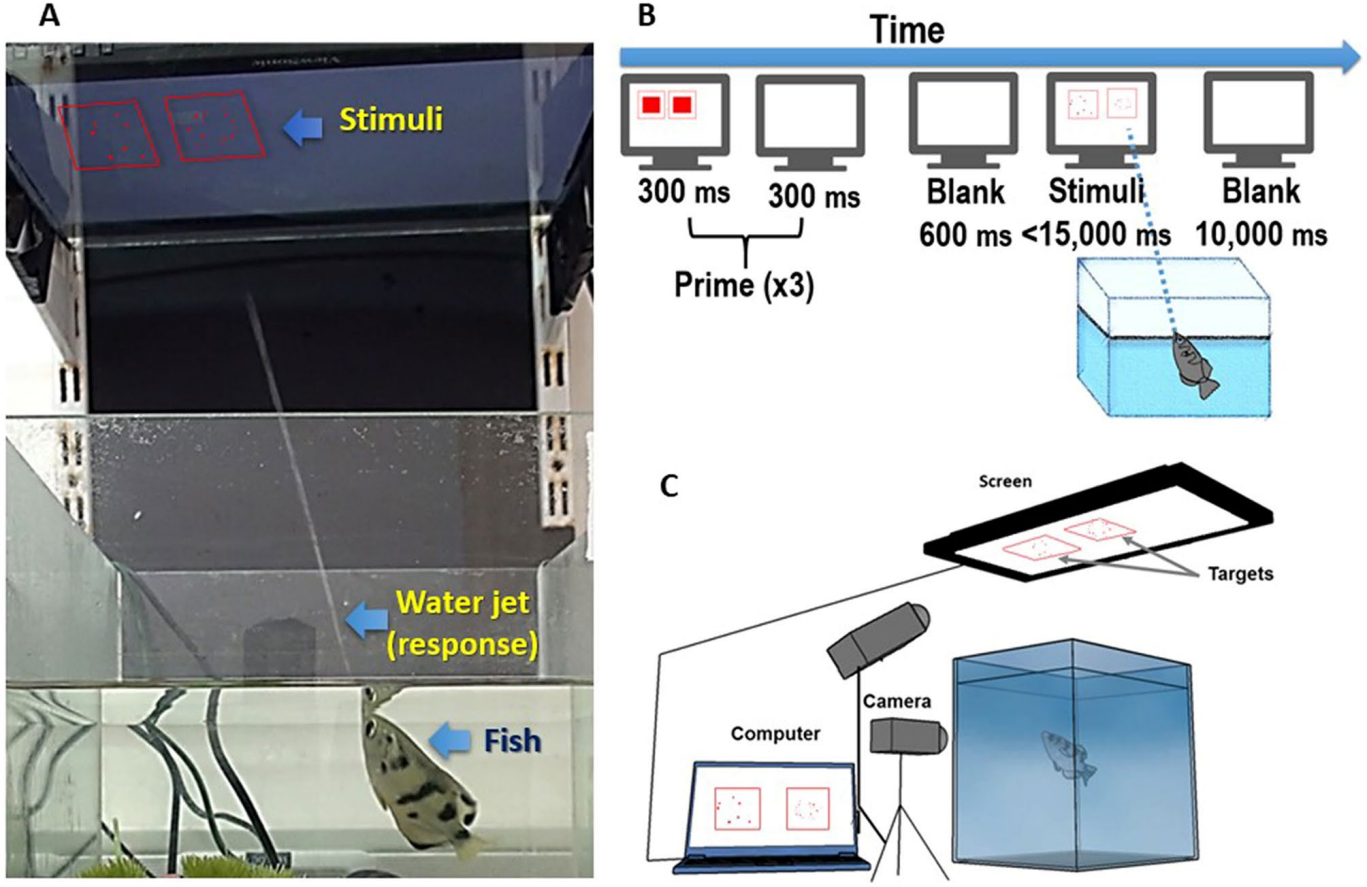
Apparatus and procedure**.** (**A**) A computer monitor is placed on a glass shelf about 50 cm from the water level. The fish respond by shooting a jet of water at one of the targets. The stimuli in this Figure are illustrations. (**B**) Procedure: a trial starts with three rapid flashes of squares in the fish’s preferred color, to attract the fish’s attention to the location where the targets will appear. Then the stimuli appear until response or until 15,000 ms have passed. Then in a 10,000 ms break, the fish is rewarded with a food pellet for responding, and the water is wiped from the glass. (**C**) The experiment was recorded by two synced high speed (120 Hz) video cameras, one camera records the fish, and the other records the screen. Part (**C**) was modified from Karoubi, Leibovich, and Segev, 2017^25^.

We separately tested eight fish. One fish (#6) was excluded from data analysis due to a failure to respond to more than 50% of the trials in every session. Each fish swam freely in a water tank during the task. The fish were previously trained to respond to targets presented on a screen positioned 50 cm above the water level (Fig. 1). Each trial started with a ‘Prime’: a flickering of two fixation boxes, indicating the place where the target stimuli will soon appear. This was done to attract the fish’s attention to the screen^24^. Six hundred ms after the prime disappeared two groups of dots appeared, each inside a frame. The fish then had to choose one of the groups by shooting water at it. The stimuli remained on screen until the fish responded or 15,000 ms have passed. After each trial, there was a 10,000 ms break in which the fish was rewarded with a food pellet if it responded, regardless of which stimulus was selected, and the screen was wiped clean of the water. The experiment was recorded with 120 frames-per-second video cameras (Fig. 1).

**Figure 2:**
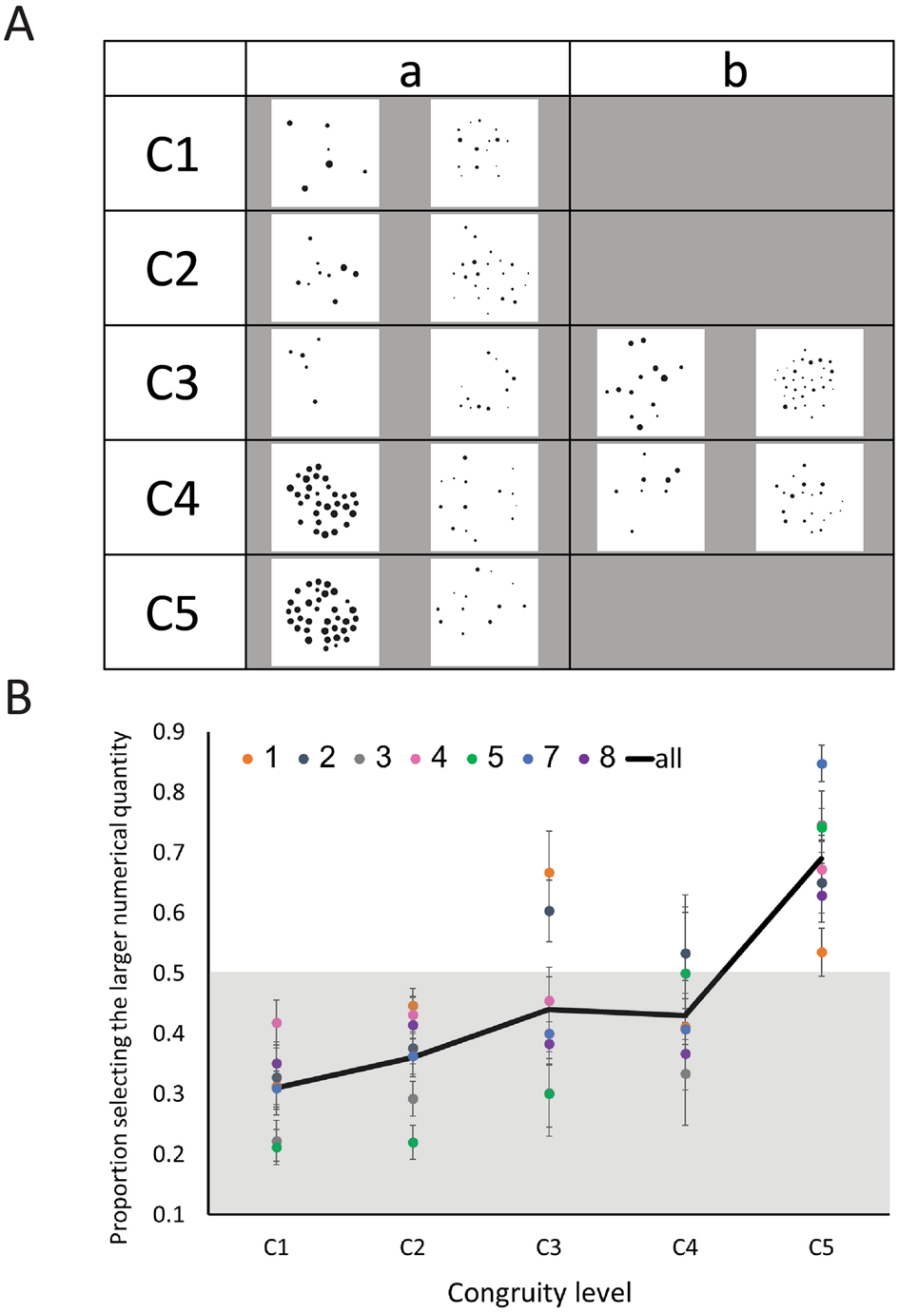
Non-numerical magnitudes influence magnitude-related decisions. (**A**) Examples for each congruity level and combination of non-numerical magnitudes. Please see Table 1 for reference as to the different combinations. (**B**) Results—the proportion of selecting the larger numerical quantity as a function of congruity level. The x-axis represents the congruity level between non-numerical magnitudes and numerical quantity. Congruity level one; only one out of five non-numerical magnitudes positively correlated with numerical quantity. The other four non-numerical magnitudes are negatively correlated with numerical quantity. Congruity level five: full congruity: all five non-numerical magnitudes positively correlated with numerical quantity. Each dot color represents one fish, and the black thick line represents the mean across fish. The gray area in the plot represents performance below chance level (for selecting the larger numerical quantity).

The stimuli the fish saw (see examples in Fig. 2a) were taken from the same stimuli set of Leibovich and Ansari^22^. There were five levels of congruity between numerical quantity and non-numerical magnitudes (Tables 1, 2). Total circumference was always congruent with numerical quantity, so there was no congruity level 0. The fish were rewarded for selecting any of the groups because the aim was to test *spontaneous* preference. Each fish completed 12 sessions (one session a day) of 40 trials each. Fish 5 completed twice the trials because we wanted to check that the lack of differences between the different combinations of non-numerical magnitudes is not non-significant due to a low number of trials. We got the same pattern in fish 5 as in the other fish, but for consistency, we analyzed only the first 12 sessions. In total each fish underwent 480 trials, 120 trials per congruity level (× 2 for Fish 5). The proportion of selecting the larger numerical quantity was tested for every fish and every congruity level.

**Table 2:** Average ratio of non-numerical magnitudes by congruity.

Congruity level	CH	AD	Den	TC	TS	Congruent magnitudes (mean ratio)	Incongruent magnitudes (mean ratio)
1	0.83	0.53	0.89	0.77	0.73	0.77	0.74
2a	0.49	0.59	0.44	0.68	0.89	0.59	0.64
2b	0.71	0.57	0.85	0.72	0.83	0.78	0.7
2 (mean)	0.45	0.58	0.65	0.7	0.86	0.69	0.67
3	0.56	0.65	0.62	0.62	0.9	0.7	0.64
4a	0.75	0.43	0.07	0.19	0.09	0.19	0.75
4b	0.9	0.68	0.91	0.59	0.83	0.81	0.68
4 (mean)	0.83	0.55	0.49	0.39	0.46	0.5	0.71
5	0.48	0.43	0.23	0.17	0.08	0.28	NA

Congruent magnitudes are the non-numerical magnitudes of the dot set that are congruent (positively correlate) with numerical quantity, and incongruent magnitudes are the non-numerical magnitudes of the dot set that are incongruent (negatively correlate) with numerical quantity. The values in the cells are the average ratio of the non-numerical ratio magnitude (smaller/larger magnitude).

*CH* convex hull, *AD* average diameter, *Den* density, *TC* total circumference, *TS* total surface area.

### Response is affected by the level of congruity

One-way ANOVA for every fish (with session number as the random factor) revealed that the proportion of selecting the larger numerical quantity significantly increased with the number of non-numerical magnitudes congruent with numerical quantity in all fish, except for fish 4 (Fig. 2). The same analysis and results were obtained at the group level (i.e., with fish as a random factor). A similar pattern of results was also observed when employing Bayesian statistics, by calculating Bayes Factors (BF) (Table 3).

**Table 3:** Results of one-way ANOVA: individual level and group level.

Fish	df	F	*p* value	η^2^ ρ	BF_10_
1	4, 32	3.89	0.011*	0.33	18.49
2	4, 36	7.46	< 0.001*	0.45	455.52
3	4, 36	16.45	< 0.001*	0.65	2.017e+6
4	4, 36	1.98	0.12	0.18	0.056
5	3, 33	17.53	< 0.001*	0.45	3,018,887
7	4, 36	20.13	< 0.001*	0.69	2.261e+7
8	4, 36	4.15	0.007*	0.32	18
All fish	4, 24	16.03	< 0.001*	0.73	94,925

The dependent measure was the proportion of choosing the larger quantity. The independent measure was congruity level (1–5).

Importantly we found that in congruity levels 1–4 selecting the larger numerical quantity was significantly below chance level, suggesting that fish preferred the group containing larger non-numerical magnitudes over the group containing a larger number of dots. This was true for all fish except fish 1 and 2 that only in congruity level 3 selected the larger numerical quantity significantly above chance level (one-tailed one-sample t-test [proportion of selecting the larger numerical quantity > 0.5] mean = 0.67, *p* = 0.004 and mean = 0.06, *p* = 0.003 for fish 1 and 2, respectively), but in congruity levels 1, 2 and 4 selected the larger numerical quantity below chance level. For fish 3–8 one-tailed one-sample t-test [proportion of selecting the larger numerical quantity > 0.5] for congruity levels 1–4: *p* > 0.24). In congruity level 5, however, all fish except for fish 2 significantly preferred the group containing the larger number of dots (one-tailed one sample t-test [proportion of selecting the larger numerical quantity > 0.5] for congruity level 5: *p* < 0.002. This pattern is also depicted in Fig. 2b.

The effect of adding congruent non-numerical magnitudes from 1–4 is linear with a shallow slope.

The plot in Fig. 2b shows a shallow slope and a linear trend up until C4, with a great change in slope in C5, which is where all non-numerical magnitudes are congruent with numerical quantity. This pattern made us ask whether the influence of the non-numerical magnitudes on performance was cumulative, or whether it is only the full congruity that influences performance. To answer this question, we calculated for each fish a fit to a linear function (R^2^) and a slope and tested whether the slopes are significantly greater than zero. The results of the individual level analysis are depicted in Table 4. All but two fish had very high fit with a linear trend. The slopes were small, and significantly different from zero (one-sample t-test against zero: t(6) = 3.75, *p* = 0.005, Cohen's d = 1.42).

**Table 4:** Slope and lienar fith for C1–C4.

Fish number	Slope	R^2^
1	0.052	0.19
2	0.037	0.7
3	0.034	0.89
4	0.026	0.89
5	0.095	0.83
7	0.034	0.93
8	0.003	0.024
Mean across fish	0.044	0.86

The slope is the ‘m’ value in the function y = mx + n. R^2^ refers to the fit to the linear function.

### The combination of non-numerical magnitudes, not their identity, contributes to the decision

The design of the stimuli allowed us to also test the influence of the identity of the non-numerical magnitudes on selecting the group containing more dots. Specifically, there were two combinations of congruity level 2 stimuli: in congruity level 2a both convex hull and total circumference were congruent with numerical quantity. In congruent level 2b density and total circumference were congruent with numerical quantity. Hence, this combination allowed us to ask whether convex hull or density carry more influence in the fish decision. (Table 1) we also had two combinations of congruity level 4. The difference between congruity levels 4a and 4b was that in congruent level 4a the average diameter of the dots was congruent with numerical quantity, and in congruity level 4b, the convex hull was congruent with numerical quantity (for the other congruent non-numerical magnitudes see Table 1). Analysis at the group level, with fish as a random factor, revealed that the proportion of selecting the group with the larger number was similar within the same congruity levels, suggesting that the influence of the identity of the non-numerical magnitudes in our data was not significant (Table 5).

**Table 5:** Comparison within the same congruity level.

Congruity level	Congruity type	Mean difference	t	*p*	BF_(1,0)_
2	a versus b	0.011	0.27	0.8	0.36
4	a versus b	0.067	0.41	0.7	0.38

The congruity types (a–b) are detailed in Table 1.

## Discussion

What do animals compare when they compare magnitudes? Our results demonstrate that when Archerfish make magnitude-related decisions, without training and without selective reward that motivates them to select one stimulus over the other, they use both numerical quantity and non-numerical magnitudes. Specifically, the chance of a fish selecting the group containing more dots moderately increases with the number of non-numerical magnitudes congruent with the number of dots and dramatically increases when all non-numerical magnitudes are congruent with numerical quantity. The identity of the specific non-numerical magnitudes that we were able to test (i.e., convex hull vs. density, and average diameter vs. convex hull), did not significantly influence performance. Our pattern of results supports the Approximate Magnitude System theory^6^, suggesting that both non-numerical magnitudes and numerical quantities are taken into account when making magnitude-related decisions, even in non-primate vertebrates. Note that up until congruity level 4, most fish selected the larger numerical quantity below chance level, and only in congruity level 4, the chance of selecting the group containing more dots was above chance level. This increase up to 4 was linear with a very moderate slope (see Fig. 2b). Only fish 1 and 2 selected the larger numerical quantity above chance level at congruity level 3. But in congruity level 4 they selected the larger numerical quantity blow chance level (and non-numerical magnitudes above chance level). Fish 1 was also the only fish that performed at chance level in congruity level 5. What can account for the differences in the patterns of fish 1 and 2?

Individual differences between fish in our data may be attributed to unknown differences in individual fish’s life history and ecology. All fish were wild-caught and acquired through pet trade, as these species do not reproduce in captivity. Furthermore, they cannot be externally sexed. We, therefore, do not know much of their individual life histories such as the population of origin, developmental history, habitat, or sex.

Another interesting finding is the moderate linear increase in the proportion of selecting the larger numerical quantity in congruity levels 1–4 relative to the spike in congruity level 5 when all non-numerical magnitudes are congruent with numerical quantity. This pattern suggests a moderate yet significant modulation of performance by the number of non-numerical magnitudes congruent with numerical quantity. The sharp increase in selecting the larger numerical quantity in congruity level 5 is also important. It suggests that it is not just having one more non-numerical magnitude congruent with the numerical quantity that is responsible for this pattern, but it may be that there are no non-numerical magnitudes that are incongruent with numerical quantity. This interpretation of the pattern supports the primacy and role of non-numerical magnitudes relative to numerical quantities, at least with this set of stimuli and in that context.

Another factor that influences performance in such tasks, is the ratio between the different magnitudes. Namely, the more dissimilar two magnitudes are, the easier it is to distinguish between them, a principle also known as Weber–Fechner law^27^. In the case of the current work, we calculated these differences as the ratio between the smaller divided by the larger magnitude. For example, the ratio of 0.1 between two densities signifies a much obvious difference compared with a ratio of 0.8. In the case of numerical quantity, we kept the ratio constant at 0.4, as we know from previous studies that in such a ratio it is easy to distinguish between two magnitudes. In the case of non-numerical magnitudes, we tried to compare the mean ratio of the non-numerical magnitudes that were congruent with numerical quantity to those that were incongruent with numerical quantity (see Table 2). In most congruity levels we reached this goal and the differences between the mean ratio of congruent and incongruent non-numerical magnitudes were minimal. Also, the ratio between the non-numerical magnitudes was always higher than 0.4, making the non-numerical magnitudes less distinguishable than numerical quantity. Yet, under these conditions, that supposedly favor numerical quantity discriminability, fish did not select the larger numerical quantity up to a full level of congruity. However, in congruity level 4, the ratio between the congruent non-numerical magnitude was 0.19 in 4a, but 0.81 in 4b. One would expect to find higher proportions of responding to the larger numerical quantity in 4b compared to 4a, but this was not the case, since the differences between performance for congruity levels 4a and 4b were not significant (Table 5). Accordingly, we suggest that in our data, the congruity and the number of non-numerical magnitudes congruent with numerical quantity were more influential than the ratio between the different magnitudes.

One of the questions that we were able to partially investigate is whether the identity or the number of the non-numerical magnitudes influences performance. Analyzing the differences between congruity levels 2a and 2b, and congruity levels 4a and 4b, allowed us to compare the influence of convex hull versus density, or convex hull versus average diameter (in congruity levels 2 and 4, respectively). We found that the identity of the non-numerical magnitudes that were congruent with numerical quantity did not affect performance. However, the BFs for these effects were very modest for (null) effect. Hence, the lack of difference within the same congruity level can be an issue of power. It is worth mentioning that to try and overcome this problem, we attempted to run twice the trials on fish 5 but got the same results with similar BF values. We would like to argue that a different study, whose aim is to create only these within-congruity-level differences, will be necessary to further explore this issue.

Our results also converge with and expand previous human studies demonstrating that the influence of non-numerical magnitudes is modulated by positive or negative correlations with numerical quantity^22,28^. Note that in magnitude comparison studies with human adults, the experimenter is always required to give some specific instructions to participants. Hence, task instructions (even vague ones) tend to focus participants’ attention on one or more stimulus properties, and hence the selection is never fully spontaneous and undirected. In humans, the closest instruction-free designs, are habituation and looking-time tasks, that are popular in studying the processing of numerical quantities e.g.,^29–31^. In such tasks, a numerical quantity is shown in different patterns multiple times and then replaced by a new numerical quantity, while the neural response or looking time is measured. If a change was detected, neural activity and looking time are expected to increase (ibid). However, in such studies, no decision is made by the participants. Moreover, the neural response to a change in magnitude does not guarantee that participants were aware of the change and would act on this change if asked to make a magnitude-related decision. In other words, a neural response can be made regardless of conscious awareness of the presented change. Fortunately, fish do not require task instructions. Hence, with our untrained fish, we were able to glance at a spontaneous decision regarding magnitudes, which is close to impossible in humans. It is our opinion that studies aiming to learn about the primacy of numerical quantities and non-numerical magnitudes in magnitude-related decisions should aspire to such un-directed performance.

The contribution of our study, compared with other studies employing spontaneous magnitude comparison tasks, is three-folds. First, we used artificial stimuli (groups of dots), without any biological relevance. This allowed us to manipulate non-numerical magnitudes more flexibly and therefore evaluate their role in the decision process in more detail. In addition, it also allows expanding the context of the task. Namely, magnitude discrimination, at least in humans, is context-dependent e.g.,^32,33^. Given the multiple tasks magnitude discrimination may be required for (hunting, mate selection, avoiding predators, shelter-seeking), it is possible that for different tasks, different magnitudes will guide an animal’s decision. Using artificial stimuli enabled us to demonstrate magnitude-related capabilities under a more general context (which is not necessarily what the animals are doing in the wild, but a proof of concept for what the animal can do, generalized across ecological constraints). With our results in mind, it would be interesting to try and use the same design with biologically relevant stimuli under different tasks, and see, for example, whether the animal’s use of magnitudes will differ while seeking shelter or hunting for prey.

Second, as mentioned in the introduction, most studies used between 1–8 items. In humans, it is debatable whether small (usually < 5) and large numerical quantities are processed by the same system e.g.,^34,35^. Since a suggestion for dichotomy exists, it is important to test the influence of non-numerical magnitudes in both small and large numerical quantities. Our study, using groups of 5–35 dots, expands the previous findings to larger magnitudes.

Third, and maybe most important, our study turns the spotlight toward non-numerical magnitudes, magnitudes that are often ‘controlled’ for but are usually not at the center of the study. As previously claimed e.g.,^6^, it is impossible to completely eliminate the influence of non-numerical magnitudes on performance in magnitude comparison tasks. This would mean to have, for example, an array of 10 dots and 20 dots that the only difference between them is their numerical quantity. Such a situation is improbable and mathematically impossible, not to mention artificial since in the environment of humans and other animals, numerical quantities and magnitudes are often confounded, correlated or anti-correlated. For these reasons, we claim that it is very important to study how non-numerical magnitudes are involved in the comparison process. The current study took this question one step forward by not only asking whether non-numerical magnitudes influence performance or not, but showing that (1) this influence, in an untrained animal, is moderated by the number of non-numerical magnitudes positively correlated with numerical quantity, and (2), that the number of non-numerical magnitudes, not their identity, influence performance. Since this is the first attempt to ask these questions in an untrained animal, and since it is well-known that the way the stimuli are built can greatly impact performance e.g.,^32,36^, replication studies with the same and different stimuli that were composed in different ways are necessary to confirm or restrict the results and conclusions of the current study. We hope that this work will inspire similar research with different animals and different types of stimuli.

Many studies in humans have demonstrated a correlation between performance and magnitude (especially number) discrimination tasks, and more sophisticated and formal arithmetic skills e.g.,^37–39^. Combining these findings with studies demonstrating that animals can discriminate magnitudes, leads to the conclusion that discrimination of numerical quantity lies at the base of arithmetic abilities in the phylogenetic sense. However, the current study adds to the body of studies providing direct evidence that in some cases animals’ magnitude-related decisions are more affected by non-numerical magnitudes than from numerical quantity, putting doubt on the claims that numerical quantity perception is the most basic building block of math.

## Materials and methods

### Subjects

Two species have been used in the study, Largescale Archerfish (*Toxotes chatareus* Hamilton, 1822) and Banded Archerfish (*Toxotes jaculatrix* Pallas, 1767). Our own data demonstrate that there is no significant difference between the two species in the performance in our tasks. The experiment was conducted in accordance with the University of Haifa’s and the State of Israel’s laws on animal care and experimentation (approval number 591/18). The experiment was performed in accordance with the relevant guidelines and regulations of Israel’s laws on animal care. All the experimental protocols were approved by Haifa University's ethics committee. Each fish was swimming freely in its tank during the task. The experiment ran on eight fish (fish #6 was excluded as mentioned in the Results section). All fish were naïve to the stimuli. All fish were previously trained to respond to a target presented on a screen placed 50 cm above the water level of their water tank. To find the fish’s preferred color, fish were presented with a red square and a black square side by side and were rewarded when responding to either one. When the fish demonstrated a clear preference to one of the colors, namely, responded over 90% of the time to the same color, the preferred color was established and used in the experiment.

### Stimuli

The stimuli for the current study were taken from the same stimuli set used by Leibovich and Ansari^22^. To fit the stimuli to fish, the background was always white, and the color of the dots was matched with the fish’s preferred color (either red or black). The dots were generated using the MATLAB code provided by Gebuis and Reynvoet^28^. Using this code, we have recorded the five different non-numerical magnitudes mentioned above. Each array contained 5–35 dots. To ensure that the fish can perceive all the visual stimuli presented, the minimum dots’ size used in the experiment was 0.2°. This is larger than the Archerfish's visual acuity, as demonstrated by their ability to detect visual structures with a minimum angle of resolution in the range of 0.075°–0.15°^40^.

### Numerical ratio

It is known that comparing two groups of dots when the numerical ratio is closer to 1 (e.g., 8 and 9) is more difficult than when the ratio is closer to zero (e.g., 6 and 30). This well-established phenomenon is known as the ratio effect e.g.^41–43^. Hence, to keep the difficulty level similar, we kept the numerical ratio constant between 0.4 and 0.42.

### Congruity levels

We manipulated the number of non-numerical magnitudes that are congruent with numerical quantity (i.e., congruity level). The congruity level ranged from 1 to 5. In congruity level 1, only one of the five non-numerical magnitudes was positively correlated (i.e., congruent) with numerical quantity, and the rest were negatively correlated (i.e., incongruent) with numerical quantity. In congruity level 5, all non-numerical magnitudes were congruent with numerical quantity (see Table 1 for more details).

We ensured that the average ratio between the congruent non-numerical magnitudes will differ minimally from the average ratio between the incongruent non-numerical magnitudes at every congruity level (Table 2). This was necessary because the ratio between the irrelevant non-numerical magnitudes might also affect performance^44^. Note that the average ratio of the congruent non-numerical magnitudes was close to the average ratio of the incongruent non-numerical magnitudes. The only exception is congruity level 4a where the average ratio of the congruent magnitude was 0.19 and the incongruent magnitude was 0.75. We discuss the implication of this difference in the discussion. Examples of the stimuli can be seen in Fig. 2a.

Each group of dots was framed with a 256 × 256 PX square in the fish’s preferred color. The squares were positioned at the same height, 192 PX apart (end to end). Both groups appeared randomly closer to the top-right, top-left, bottom-right or bottom-left corners of the screen. In total, we had 240 pairs of dot arrays: 60 for each congruity level (1–5). In 50% the larger numerical quantity of dots appeared on the right-hand side of the screen. The stimuli were divided into six blocks of 40 stimuli each so that each block contained 10 trials per congruity level.

### Apparatus

The experiment ran using OpenSesame 3.1^45^, on a Windows 7 computer. The screen resolution during the experiment was set on 1024 X 768 px. The display was duplicated onto a 21-inch screen placed 42 cm above the water tank. Two synchronized high-speed cameras recorded the fish and the screen at a rate of 120 Hz. The videos were used to verify the fish’s response.

### Procedure

Each session included one block of 40 trials. Each fish was tested in 12 sessions (5 sessions per week, for two weeks) to provide a total of (12 × 40 =) 480 trials. The order of the trials within the block was random. Each trial began with a ‘prime’: a flashing pair of squares aim to attract the fish attention to the location of the target to come. The squares (128 × 128 PX each) appeared within a 256 × 256 frame for 300 ms and disappeared for 300 ms three times. After a blank screen showing only the frames, dot arrays appeared where the location of the flashing squares appeared before. The dot arrays disappeared once the fish responded or when 15,000 ms have passed. Between each trial, there was a 10,000 ms blank screen. At this time, the fish was rewarded with a food pellet (for responding to any of the dot arrays) and the screen was cleared of water by the experimenter. Each session lasted about 12 min.
